# Disaster Impacts Surveillance from Social Media with Topic Modeling and Feature Extraction: Case of Hurricane Harvey

**DOI:** 10.1007/s13753-022-00442-1

**Published:** 2022-09-23

**Authors:** Volodymyr V. Mihunov, Navid H. Jafari, Kejin Wang, Nina S. N. Lam, Dylan Govender

**Affiliations:** 1 Department of Environmental Sciences, Louisiana State University, Baton Rouge, LA 70803 USA; 2 Department of Civil and Environmental Engineering, Louisiana State University, Baton Rouge, LA 70803 USA; 3 Division of Electrical and Computer Engineering, Louisiana State University, Baton Rouge, LA 70803 USA

**Keywords:** Disaster impacts, Hurricane Harvey, Infrastructure impacts, Latent Dirichlet allocation (LDA), Social media analysis, Twitter data

## Abstract

Twitter can supply useful information on infrastructure impacts to the emergency managers during major disasters, but it is time consuming to filter through many irrelevant tweets. Previous studies have identified the types of messages that can be found on social media during disasters, but few solutions have been proposed to efficiently extract useful ones. We present a framework that can be applied in a timely manner to provide disaster impact information sourced from social media. The framework is tested on a well-studied and data-rich case of Hurricane Harvey. The procedures consist of filtering the raw Twitter data based on keywords, location, and tweet attributes, and then applying the latent Dirichlet allocation (LDA) to separate the tweets from the disaster affected area into categories (topics) useful to emergency managers. The LDA revealed that out of 24 topics found in the data, nine were directly related to disaster impacts—for example, outages, closures, flooded roads, and damaged infrastructure. Features such as frequent hashtags, mentions, URLs, and useful images were then extracted and analyzed. The relevant tweets, along with useful images, were correlated at the county level with flood depth, distributed disaster aid (damage), and population density. Significant correlations were found between the nine relevant topics and population density but not flood depth and damage, suggesting that more research into the suitability of social media data for disaster impacts modeling is needed. The results from this study provide baseline information for such efforts in the future.

## Introduction

With the rapid development of mobile technology, social media have transformed human interactions. Researchers increasingly utilize social media analysis to propose innovations in disaster resilience research (Huang and Xiao 2015; Imran et al. 2015; Wang and Ye 2018a). Several studies confirmed the pivotal role of social media use in disaster resilience of the affected communities (Zou et al. 2018, 2019; Wang et al. 2019; Wang et al. 2021). For instance, Zou et al. (2019) analyzed Twitter and social-geographical data during the 2017 Hurricane Harvey and found that the density of disaster-related tweets in a county was related to its better social-geographical conditions. Similar assessment for Hurricane Isaac by Wang et al. (2021) confirmed these results, suggesting that higher Twitter use can improve resilience of communities.

**Fig. 1:**
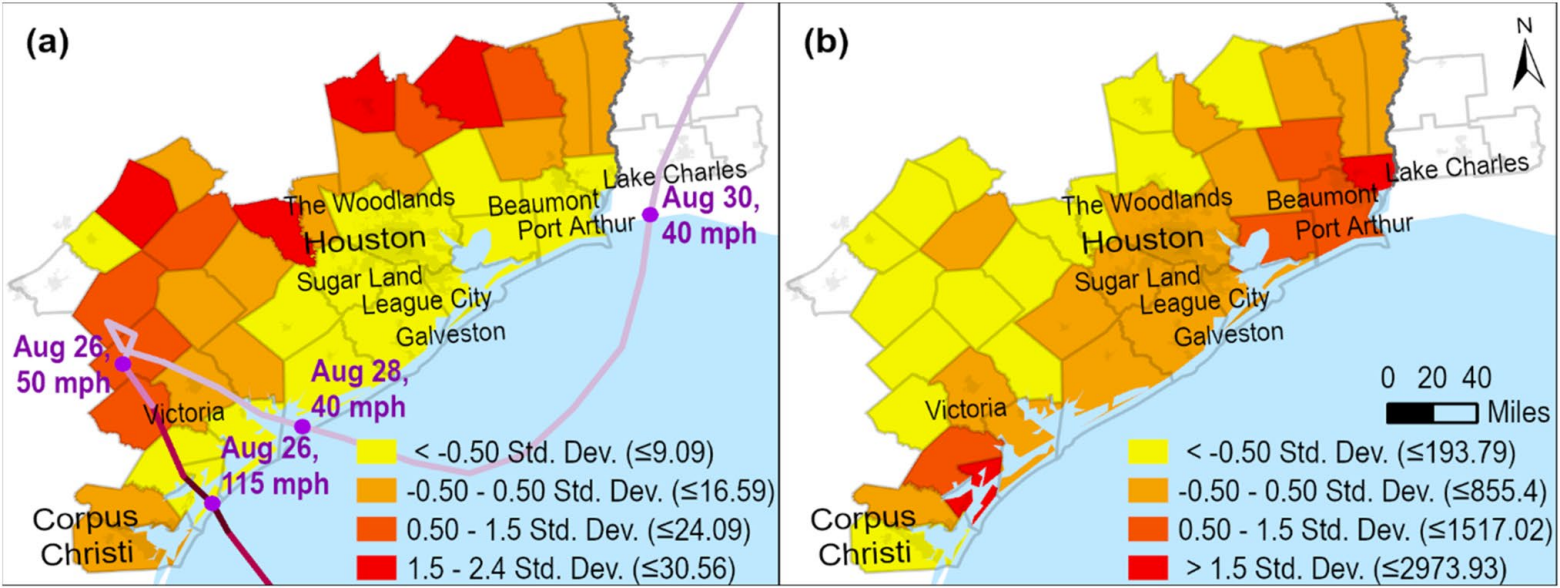
Hurricane Harvey county-level **a** average flood depth (inches) (FEMA 2018), and **b** distributed disaster aid (USD per capita) (FEMA 2020)

Hurricane Harvey is one of the first major natural hazard-related disasters in the United States with widespread adoption of social media by the public and to this day serves as an important case for studying social media use during catastrophic events (Mihunov et al. 2020). It hit the Texas coast near Corpus Christi as a category 4 force on 26 August 2017, quickly slowed down, and changed direction moving to the Houston metropolitan region. Harvey subsequently delivered over 1.5 m (5 ft) of rainfall (Watson et al. 2018), causing unprecedented flooding and damage (Fig. 1).

Among the tasks of emergency disaster response is rapidly surveying and assessing the impacts and damage to vital civil and social infrastructure. During a disaster, people report through social media in real time to share valuable information with their neighbors (Imran et al. 2015; Jamali et al. 2019; Li et al. 2019). Potentially, this information can be used by emergency operations managers. Because social media data are unstructured, large volume, and presented in natural language, using them for informing rapid disaster response remains challenging. Natural language processing (NLP) could address these challenges, as it combines computational linguistics, computer science, and artificial intelligence to enable information extraction from natural human speech, that is, text mining (Russell and Norvig 2010; Sarkar 2016; Albalawi et al. 2020).

Topic modeling was used in this study to understand the content of Twitter data collected during Hurricane Harvey and extract the information related to impacts and damage to infrastructure. Topic modeling is a term describing a set of text mining methods, which are used to detect hidden thematic structures in extensive collections of documents through unsupervised machine learning (Blum et al. 2020). This approach is needed because the size of social media data prohibits manual labeling and sorting (Wang and Ye 2018a).

The objective of this study is to quantify discussions of infrastructure impacts or damage from the tweets by filtering out noisy irrelevant content and evaluating their suitability for modeling real-world impacts. More specifically, our research questions are: (1) Do the discussions during a major disaster contain infrastructure-related information that should be of alert to emergency management; (2) How do the topics of discussion related to infrastructure impacts change over the course of the disaster response; and (3) Does the spatial pattern of the locations of infrastructure-related tweets correlate with other measurements of real-world phenomena, such as flood depth, distributed disaster aid, or population density. While topic modeling has been previously tested on social media data in a disaster context (Alam et al. 2020; Ferner et al. 2020; Xu et al. 2020), research questions regarding infrastructure impacts information on social media remain unaddressed. Answering these questions will inform practical applications of mining social media data to improve response and recovery. This approach can be applied in future disasters to extract relevant information in near real-time for emergency response and management (Ferner et al. 2020; Yao and Wang 2020).

## Background

Social media are increasingly becoming a focus of disaster resilience research (Huang and Xiao 2015; Wang and Ye 2018a; Zou et al. 2018). Studies have been made in investigating the role of social media in situational awareness, the type of dynamic decision making founded in one’s continuous perception and comprehension of changing elements of the environment and ability to make accurate projections of future changes (Endsley 1995). The methods include categorizing or quantifying the contents of the disaster-related messages (Huang and Xiao 2015; Imran et al. 2015; Wang and Ye 2018b; Alam et al. 2020). According to Imran et al. (2015), the disaster-related social media content can be categorized based on emotional substance or neutrality, information source, credibility, location, time of the message, and the type of information provided. Moreover, the factual or useful information in the tweets can be related to caution and advice, affected people, infrastructure and utilities, needs and donations, miscellaneous (Imran et al. 2015), as well as weather and environment (Wang and Ye 2018b). Building on previous findings that large volume of infrastructure-related messages is posted during disasters (up to 50% of all useful posts according to Wang and Ye 2018b), which have not been a focus of a dedicated study, our goal is to further investigate the content of these messages, thus addressing a research gap.

Previously social media have been evaluated for suitability to act as a “social sensor” and assist in early damage predictions. For example, Kryvahseyeu et al. (2016) found positive correlations between damage represented by Federal Emergency Management Agency (FEMA) household disaster assistance and Twitter activity during Hurricane Sandy. Similarly, Samuels et al. (2020) studied the 2017 Atlantic Hurricane season (Harvey, Nate, Irma, Maria) and found correlations between the magnitude of deviations (sharp increases and decreases) of the Twitter activity and a discrete FEMA damage assessment indicator. These results are promising, but limitations of this approach remain, such as social-geographical representativeness of Twitter users (Zou et al. 2019; Wang et al. 2019), accuracy and availability of location information (Middleton et al. 2018; Wang et al. 2021), and aggregation of all tweets regardless of their content (Wang and Ye 2018a).

Text mining and specifically topic modeling of social media data presents its own set of challenges as the data consist of short messages, as opposed to long documents such as paragraphs in articles and book chapters (Cheng et al. 2014; Albalawi et al. 2020). Albalawi et al. (2020) compared the performance of several topic modeling methods on short text data and found that latent Dirichlet allocation (LDA) (Blei et al. 2003) showed greater topic coherence and overall model interpretability than other methods. In this study, we used LDA as a readily available and widely used method for topic modeling.

Several studies demonstrated LDA applications with the Twitter data collected during major disasters. For example, Alam et al. (2020) used LDA to detect general discussion topics in Twitter data during Harvey, Irma, and Maria. Similarly, Xu et al. (2020) applied LDA to Twitter data at different disaster stages of Irma, thus describing general topics of public discussion that varied from news to local updates and advice, to political discussion. On the other hand, Ferner et al. (2020) proposed a modification of LDA that initializes with automatically generated seed words and demonstrated that it improved topic coherence on Hurricane Harvey and Napa Valley earthquake Twitter data. In another application, Yuan et al. (2021) mapped LDA-derived topics of social media discussion during Hurricane Florence to the demographic characteristics of users that disclosed their full names on Twitter, revealing differences between genders and ethnicities. More recently, Xue et al. (2020) used LDA topic modeling for exploring the topics of discussion and assigning their dominant sentiment during the early onset of COVID-19 pandemic. Similarly, Lyu and Luli (2021) conducted a study using tweets from March to August 2020 to examine tweets related to official Centers for Disease Control and Prevention (CDC) messaging. They found that the themes of disease mortality and credibility of the CDC guidance were prevalent.

Unlike the previous disaster literature that described general Twitter discussion using LDA, our study pursues its practical application by focusing specifically on information relevant to disaster impacts and damage assessment of infrastructure. This study aims to meet the demand for an efficient and accessible approach to extracting infrastructure damage and impact reports from social media, thus making their data useful for emergency managers, who operate with scarce time and resources. In this study, we apply several filtering steps and LDA topic modeling to subset the Twitter data specifically related to the performance of civil and social infrastructure. An unsupervised method is especially advantageous since pre-labeled tweets are rarely available and time-consuming to obtain. The topics produced by LDA are then used to further categorize and describe the types of infrastructure-related information found in the Twitter data. This study is among the first to detect and quantify infrastructure impact messages in social media data; it provides baseline information and a training dataset for improving text mining and object recognition models for damage detection in the future.

## Data and Methods

**Fig. 2:**
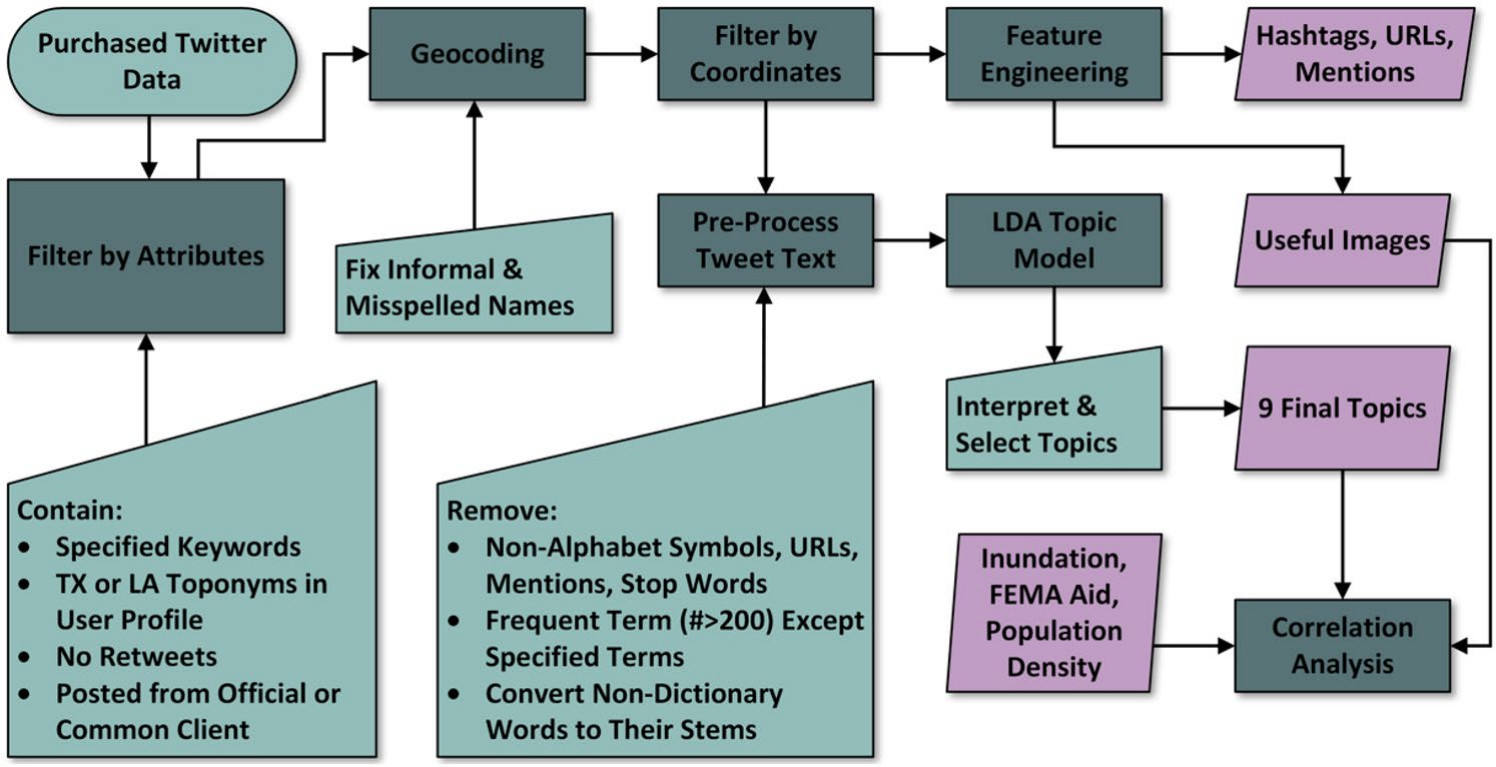
Flowchart of the steps and procedures for social media analysis with topic modeling and feature extraction for infrastructure impacts mining

This section describes the steps taken that include data acquisition and filtering, pre-processing of the text data, LDA modeling, feature extraction and content analysis, and analysis of spatial and temporal patterns of the extracted topics (Fig. 2).

### Data Acquisition and Filtering

We utilized our Hurricane Harvey dataset (17 August to 7 September 2017) previously purchased from the Twitter company using 21 keywords “hurricane, harvey, disaster, cajun navy, hurricaneharvey, txdps, txtf1, redcross, coastguard, houstonpolice, houstonoem, salvationarmy, flood, sos, flooding, storm, rescue, sendhelp, cajunnavy, fema, salvation army.” The total number of tweets in the dataset is approximately 45 million. Several steps were taken to select a smaller sample of tweets relevant to this study on infrastructure damage. The Twitter data are stored in JavaScript Object Notation (JSON) format, and we utilized *tidyjson* package in *R* (Stanley and Arendt 2020) to access and manipulate the data (for example, extract information from JSON fields).

The first step involved filtering by infrastructure-related keywords,^1^ which yielded a subset of 12,316,629 tweets. We then proceeded filtering the resulted subset using regular expressions to select tweets from users having Texas or Louisiana as profile locations. In this procedure, we used all the common spellings, including informal spellings and abbreviations of the toponyms from Texas and Louisiana frequently found in the data to accomplish this task. This was carried out to avoid geocoding the data that will be later discarded. These operations yielded a subset of 1,373,625 tweets presumed to be specific to Texas and Louisiana geographically. To extract first-hand information (Imran et al. 2015), we further removed retweets and only kept original tweets, which yielded a subset of 311,068 tweets. To select the tweets that were not generated automatically, we selected only those that were posted using several official or common un-official Twitter clients. The geocoding of the resultant subset was accomplished using *tidygeocoder* package in *R* (Cambon et al. 2021). We generated coordinates for each tweet by using user profile location information with Census Geocoder (U.S. Census Bureau 2022) and Google Geocoding APIs (Google 2022) in *tidygeocoder*. We iteratively checked for errors or unassigned coordinates in the output and manually replaced misspelled or informal toponyms to correct them, until all the tweets were assigned locations with a reasonable certainty. The final filtering step discarded the tweets from the users with profile locations outside of the area affected by Hurricane Harvey (Fig. 1), which narrowed down the sample size for text mining to 127,944 records.

### Pre-processing of the Text Data

In text mining, individual words are commonly referred to as terms, and a set of words is called a document (Blum et al. 2020). Documents are then organized into corpus, which is a data structure used for machine learning from natural language. In our study, the Twitter data were converted into a corpus structure, with each tweet representing an individual document. For simplicity, we used unigrams as document terms, meaning a term can consist of one word only. We implemented pre-processing procedures commonly prescribed in LDA topic modeling. First, punctuation, non-alphabet symbols, links, mentions, and numbers were removed. Then, we identified and removed a list of stop words based on several stop-word lists^2^ and highly frequent terms (over 200 occurrences in the model, with informed exceptions). We carried out this step because common non-descriptive words and many frequent terms in the training data can worsen the model performance and make interpretation difficult (Schofield et al. 2017; Fan et al. 2019). Additionally, terms used no more than once in the entire corpus were compared with Grady Augmented dictionary from package *qdapDictionaries* (Rinker 2013), thus allowing us to remove non-dictionary words. These procedures are similar to those recommended in Grün and Hornik (2011) to avoid terms that are too frequent or too rare in the vocabulary. The terms were stemmed (converted into their root) using a Porter’s stemming algorithms implemented in the *tm* package in R (Feinerer and Hornik 2020). Finally, documents (tweets) with less than three terms (words) were removed from the corpus. After all these steps, the final size of the dataset used in the topic modeling is 106,710 documents (tweets).

### LDA Modeling

The LDA introduced by Blei et al. (2003) is a widely used topic model that is considered more complete and an improvement over previous latent semantic allocation (LSA) models (Phan et al. 2008). It is an unsupervised algorithm within the Bayesian statistical paradigm, which assumes that latent topics exist within the data where each topic is a probability distribution over words (Chakkarwar and Tamane 2020; Lyu and Luli 2021). Unlike typical clustering (for example, k-means) that assumes a distance measure between clusters and assigns each data point to a particular group, topic modeling produces probabilities of a document belonging to several topics (Imran et al. 2015; Blum et al. 2020; Lyu and Luli 2021). Among the advantages of LDA are its ability to produce a set of individually understandable topics from a large size corpus without the need for pre-labeling or prior knowledge, and its ability to handle mixed-length documents (Albalawi et al. 2020). However, LDA requires a predefined number of topics, which comes with a trade-off. Specifying a smaller number of topics tends to produce more general categories, whereas overlapping categories and themes are a likely result of using a larger number. The number of topics is usually selected by fitting many models and selecting the one with a better performance, such as in terms of perplexity or log-likelihood (Griffiths and Steyvers 2004; Grün and Hornik 2011). We used Griffiths and Steyvers’ marginal likelihood metric implemented in the *ldatuning* package (Griffiths and Steyvers 2004; Murzintcev and Chaney 2020) to find the “optimal” number of topics and Gibbs sampling implementation of LDA in the *topicmodels* package (Phan et al. 2008; Grün and Hornik 2011) to estimate the final model.

### Feature Engineering and Content Analysis

After assigning the posterior topics to each tweet, we extracted additional features from the Twitter data to better understand their content within each topic. These features are hashtags, mentions, and URLs that users include in their tweets. Hashtags were converted to lower case, and URLs were shortened to their domains to find frequently linked websites, rather than their individual pages. To focus on the features that appeared in the data frequently, and avoid revealing private information, we will mention only those that appeared at least 200 times in the tweets from the final topic model.

A user can attach up to four images to a tweet. When the tweets contained images, we downloaded them using direct links supplied with the Twitter data. We then used the *ImageIdentify* function in *Wolfram Mathematica* to process those images (Wolfram Research, Inc. 2021). *ImageIdentify* is a fully trained neural network that returns the most likely object depicted in the image with a set specificity (a value from 0 to 1) and probability acceptance threshold (chosen automatically by default). We used this function to identify common types of images that were not related to disaster damage in the dataset, such as images with text, screenshots, images of TV screens and signs, images of people and pets not related to the disaster damage, among others. We used maximum specificity and then manually grouped many recognized objects into more general categories (Ford 2017). After these features were extracted from the data, they were cross tabulated with the LDA topics.

### Analysis of Spatial and Temporal Patterns

To further investigate how the Twitter topics relate to the real world, we analyzed their temporal and spatial patterns. For the temporal analysis, we tabulated the infrastructure-related topics by date, and investigated the differences in their frequencies across the span of the disaster. For the spatial pattern analysis, we tabulated the tweets in each topic, as well as tweets with images in each topic, per county and per 10,000 population in that county, according to their geocoded locations. Then the average county inundation was tabulated by authors from FEMA rainfall depth raster (FEMA 2018) in ArcGIS Zonal Statistics tool, excluding the pixels with no data (Esri 2021). We then correlated the county-level Twitter data with the average flood depth, damage per capita (FEMA 2020), and population density (per square kilometer) (U.S. Census Bureau 2021), to understand if the features from Twitter resemble the patterns of these real-world phenomena.

## Results

The results of our study discussed in this section include posterior topics, relevant topic selection, analysis of the extracted features (hashtags, mentions, URLs, and useful images), as well as county-level correlation analysis of the useful topics and images with flood depths, disaster aid (damage), and population density (Fig. 2).

### Latent Dirichlet Allocation (LDA) Topic Model

The LDA modeling results reveal 24 topics along with their most probable terms. The summary descriptions of the topics were created by examining both the top-terms from the model output, and the content of at least 100 tweets that were randomly selected from each topic. We grouped the topics into four categories. The first three groups were (1) Casual discussions; (2) News, updates, politics, and viral topics; and (3) Disaster relief discussions (Table 1).

**Table 1:** Latent Dirichlet allocation (LDA) topics excluded from further analysis, along with their most probable terms

	Topic	Terms	#	%
Casual conversations				
1	Closures, interruptions, delays	school, suppli, eclips, hisd, sept, aug, delay, colleg, teacher, footbal	6378	5.98
4	Time with family, Astro’s game	hous, name, astro, wife, son, throw, ladi, tough, spend, win	5278	4.95
10	Prayers and good wishes	cross, red, faith, american, prayersfortexa, peac, spirit, dear, trust, power	4435	4.16
15	Worry about warnings	flash, emerg, inch, street, phone, power, issu, servic, thunder, lightn	4075	3.82
23	Inconvenience, boredom	hous, stuck, bore, tire, swear, bruh, imma, alright, bed, smoke	3940	3.69
News, updates, politics, viral topics				
5	Political figures and events	obama, shoe, black, idiot, club, wear, gov, abbott, sandi, vote	4791	4.49
13	Hurricane-related viral topics	evacu, osteen, funni, spread, facebook, lakewood, luck, sens, type, dirti	4184	3.92
16	Meteorological events	damag, mile, depress, unpreced, strengthen, trillion, stall, inch, dump, slow	3934	3.69
17	Dangers of floodwaters	fire, sky, ant, gator, snake, don, light, blue, nasti, allig	3096	2.90
20	Need for climate investment	system, build, energi, wall, infrastructur, dam, climat, develop, reservoir, prevent	3459	3.24
Disaster relief discussions				
9	Evacuation issues	evacu, hospit, road, issu, voluntari, patient, traffic, judg, emerg, mass	5236	4.91
3	Shelter calls for volunteers	shelter, anim, pet, evacu, volunt, rescu, suppli, fear, program, deport	5267	4.94
14	Volunteer rescue updates	rescu, volunt, cajunnavi, train, emerg, app, repost, harveyso, clock, harveyrelief	3718	3.48
18	Cleanups, hot food, water	power, bottl, cleanup, debri, hot, bag, suppli, trash, ice, wine	3719	3.49
24	Fundraising for recovery	organ, campaign, gofundm, music, shirt, chariti, song, concert, fan, join	3032	2.84

**Fig. 3:**
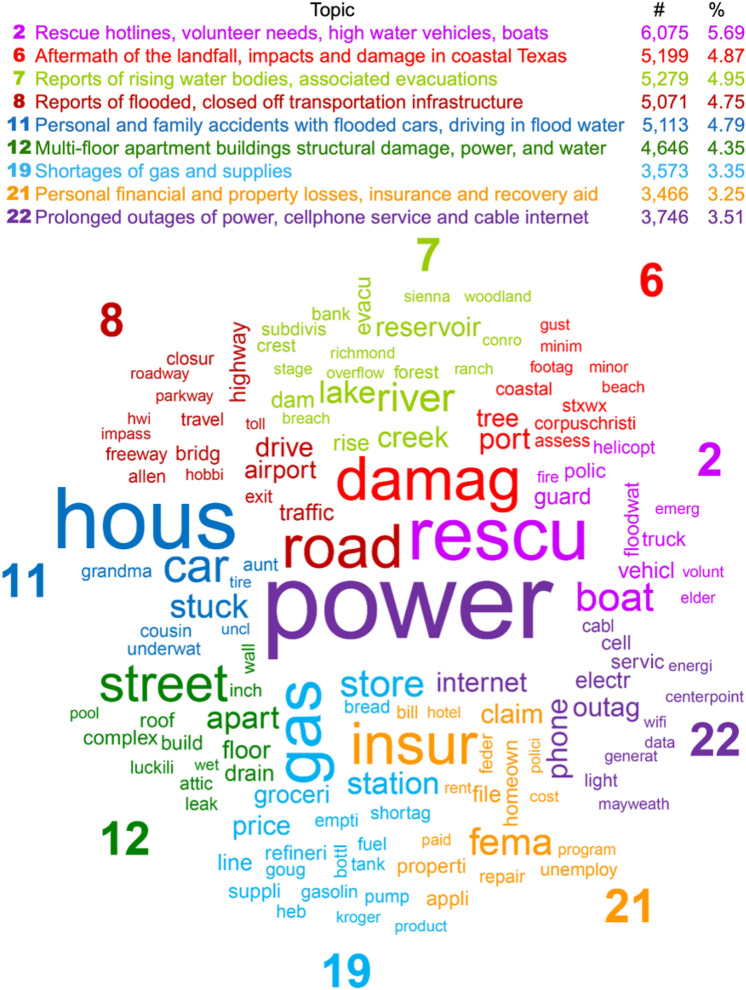
Comparison word cloud for the nine infrastructure-related topics. Word size corresponds to its deviation from the group mean, and color and placement corresponds to each topic.

Data in these categories are useful for various applications—for example, groups 1 and 2 can help in analyzing public sentiment, as well as the spread of information from valid sources or misinformation. Group 3 can help in supplying additional source of data for better coordination of relief and recovery efforts, or various other uses. However, the focus of this study is on the topics in group 4, which are related to infrastructure impacts and damage. We refer to them as the nine infrastructure-related topics, and Fig. 3 presents them as a comparison word cloud (Fellows 2018). The word cloud shows that each topic exhibits its own distinct theme, despite small overlaps. The topics are reports of high water, and local needs for rescue vehicles and equipment (T2), aftermath of the landfall, impacts and damage in coastal Texas (T6), overflowing waterbodies, and associated evacuations (T7), road and transportation impacts (T8), vehicle accidents and impacts (T11), gas and supply shortages (T19), insurance and assistance claims for property damage (T21), as well as power and Internet outages (T22), all related to the events of Hurricane Harvey.

### Content Analysis

To provide more context on the contents of the tweets in each of the nine topics, we discuss the frequent hashtags, linked websites, mentioned users, and images and how frequently they occur. The numbers listed in this section represent the percentage of tweets in the topic that contain a particular feature (URL, mention, hashtag, or an image category). Total number of tweets in each topic are listed in Fig. 3.

For example, we found 25.2% of all tweets (*n* = 106,710) had links to other tweets (twitter.com URLs), which is an equivalent of a “quote tweet” or a retweet with a comment added by a user. This is the highest share compared to other types of links in the data. Transportation infrastructure impacts (T8) topic held the highest percentage of links to other tweets (29.4%) among all topics, which is likely due to users more actively re-sharing the information on closed roads and flooded streets to alert others. Similarly, we found a high percent of quote tweets (27.43%) in the topic of high water in inland lakes and rivers, and associated evacuation orders issued by local governments (T7). Second highest service linked in the tweets was Facebook (fb.me domain, which is used in automatic cross-posting from Facebook to Twitter). The highest percent of Facebook cross-posts was in the property damage, insurance, and government assistance topic (T21 with 10.8%), likely due to many details requiring longer posts than Twitter allows. Other topics with frequent Facebook links were that of impacts and damage to coastal areas of Texas (T6, 7.3%), and urban flooding and rescue needs (T2, 5.8%). In these topics, users may attempt to rapidly distribute the information, so they posted on multiple platforms. Next was Instragram (instagram.com), a social media platform usually is for posting images and videos, that was more frequently linked in the topics of overflowing waterbodies (T7, 4.1%) and multiunit housing impacts (T12, 3.9%). There were also two local news sources (chron.com and houstonchronicle.com) and Periscope (pscp.tv), a service for live-streaming (now inactive). Periscope was the most linked in the topic of coastal impacts (T6, 1.2%), which is likely due to users sharing links to live cameras streaming from the places of the hurricane landfall. Facebook also has a live-streaming feature, which might explain the high percentage of Facebook links (7.25%) in this topic. As for the most mentioned Twitter users, we found local newspaper @HoustonChron in the topic of gas and supply shortages (T19, 1.8%), and local news organizations such as @abc13houston in the topics of issued evacuations (T7, 2.3%) and rescue needs (T2, 2.01%), as well as @KPRC2 and @KHOU in the topic of evacuations (T7, 2.1% and 1.6%, respectively). This shows that Houston residents were actively engaging with local news organization, both relying on information from the news and supplying updates to the news organizations as well. This further proves as a model that emergency operation centers can not only disseminate but also receive updates on infrastructure impacts. Houston Police (@houstonpolice) was the most mentioned in the topic of search and rescue needs (T2, 1.25%); meteorologist for Harris County (@JeffLindner1) was the most mentioned in the topic of flooding waterbodies and evacuations (T7, 1.52%); and FEMA (@fema) was the most mentioned in the topic of government assistance and property damage claims (T21, 1.24%).

As for the hashtags, their use was quite frequent, but they are more general, and were likely used to separate the discussions of Hurricane Harvey from other topics on Twitter, rather than organize the discussions of the Hurricane Harvey into more concrete sub-discussions within itself. The hashtags mentioning Houston (#houston, #houstonflood, #houstonstrong) may help separating the tweets from those coming from other affected areas because they are more frequently used in the topics affecting Houston and inland areas, such as rescue needs (T2), evacuations (T7), and transportation disruptions (T8). #rockport was the most used, as expectedly because of the hurricane landfall location, in the topic of coastal damage (T2, 1.88%).

Overall, low percentage of links, frequent mentions, and hashtags is desirable in our data analysis because it indicates that the data identified by the topic modeling contain more of first-hand accounts, rather than re-shared information, or posts from people not involved in the disaster.

On another hand, tweets containing images are highly desirable, as many of them contain photographic evidence of disaster damage and impacts on infrastructure, such as floodwaters, debris, flooded houses, vehicles, fallen trees, and poles. The *ImageIdentify* function in Wolfram Mathematica is not trained to reliably recognize these categories, but it can recognize common objects. We used this ability to help us filter out irrelevant images, such as text, graphics, maps, screenshots, people, pets, among many others. This left us with mostly useful images that we separated into three categories based on the objects that *ImageIdentify* could reliably recognize. They were landscapes, which were mostly photographs of floodwaters and flooded streets; vehicles, which included flooded cars, high-water vehicles, and sometimes boats and vessels; and artefacts, which were various objects such as debris, fallen trees, and flooded houses, among others. This approach for image classification is quick and readily available, but it is limited to one category per image, that the algorithm finds the most probable. Better models are needed to recognize multiple objects and identify their relevancy in disaster-related photos reliably, thus enabling more robust algorithmic damage evaluation (Jafari et al. 2021).

When cross-tabulating image categories with the topics, we found many images specifically related to the topic recognized by the LDA. Moreover, most of the infrastructure-related topics had higher than the overall average percent of tweets with images, as well as images identified as useful. For example, T8 had 5.9% of tweets with “landscape” images, most of which were photographs of floodwaters and flooded streets, and similarly 5.1% of “landscape” images in T7 depicted many photos of flooded rivers and lakes. We show several examples of tweets, chosen at random, from each of the infrastructure-related topics in Table 2, including the tweets with images and their corresponding recognized categories. They are examples of tweets that either describe or depict disaster impacts and can be used in early damage estimation.

**Table 2:** Tweet examples in infrastructure-related topics. Image categories captioned in cursive.

Tweet					Date	T#
@KHOU High water rescue needed at <address> elderly couple with chest deep water in home					08-27	2
FLOODING: @HoustonFireDept trying to make water rescues on 610. @Fox26Houston #Harvey					08-27	
West of #Houston, steady #rain, #flashflooding & overnight winds lead to damaged trees. #HurricaneHarvey					08-26	
@uscoastguard Residents of Port Arthur TX are taking on major water. And Some have lost power. Please send or alert Help.					08-30	
There was a lot of flooding around the Lake Houston and San Jac River area. Just wanting to bring awareness. @KPRC2					09-01	7
@abc13houston Shadow Creek Ranch along Kingsley Dr in Pearland under water #houstonflood #HoustonStrong					08-29	
East Sam Houston Tollway SB Spencer exit closed due to flooding on service road verified 5:10 a.m.					08-27	8
Eerie scene in Port Arthur. Dozens of cars parked along higher streets to avoid flood waters					08-28	
While y’all are making jokes about #hurricaneharvey my house is being flooded out & my car is underwater. This isn’t a joke people.					08-27	11
My aunt’s house done started to flood now and we stuck here with 3 dogs					08-30	
Doing great. Thankful we didn’t flood or get hit with a tornado. Still no power. Street water still high but draining thankfully.					08-27	12
Cars in my apartment complex – mine and <mention> included – might get lost to this flooding. #SETXNews #Harvey					08-27	
You should have seen @Costco gas station tonight. It was crazy with lines down the street. #HurricaneHarvey #houston					08-24	19
Houston Shell Mart@Wallisville/Beltway8 open #houstonflood #Houston #store #gas #HurricaneHarvey #food #HurricaneHarvey #flood #storm #rain					08-28	
FEMA will not be covering any of the financial damages to my property because, even though I LIVE THERE, it’s zoned as “commercial”.					08-31	21
We are registered with FEMA. In case of emergency or evacuation reach us at <phone#>. @VisitPortArthur @gpacc1 @port @Qualityinn_po					08-29	
...and the power is out #HurricaneHarvey #HoustonTX #SpringTX					08-26	22
@ATT hasn’t restored internet to us yet, that Hurricane was no joke. At this rate I’ll be playing @DestinyTheGame on a Hotspot. #FeelsBadMan					09-02	

### Temporal Patterns

**Fig. 4:**
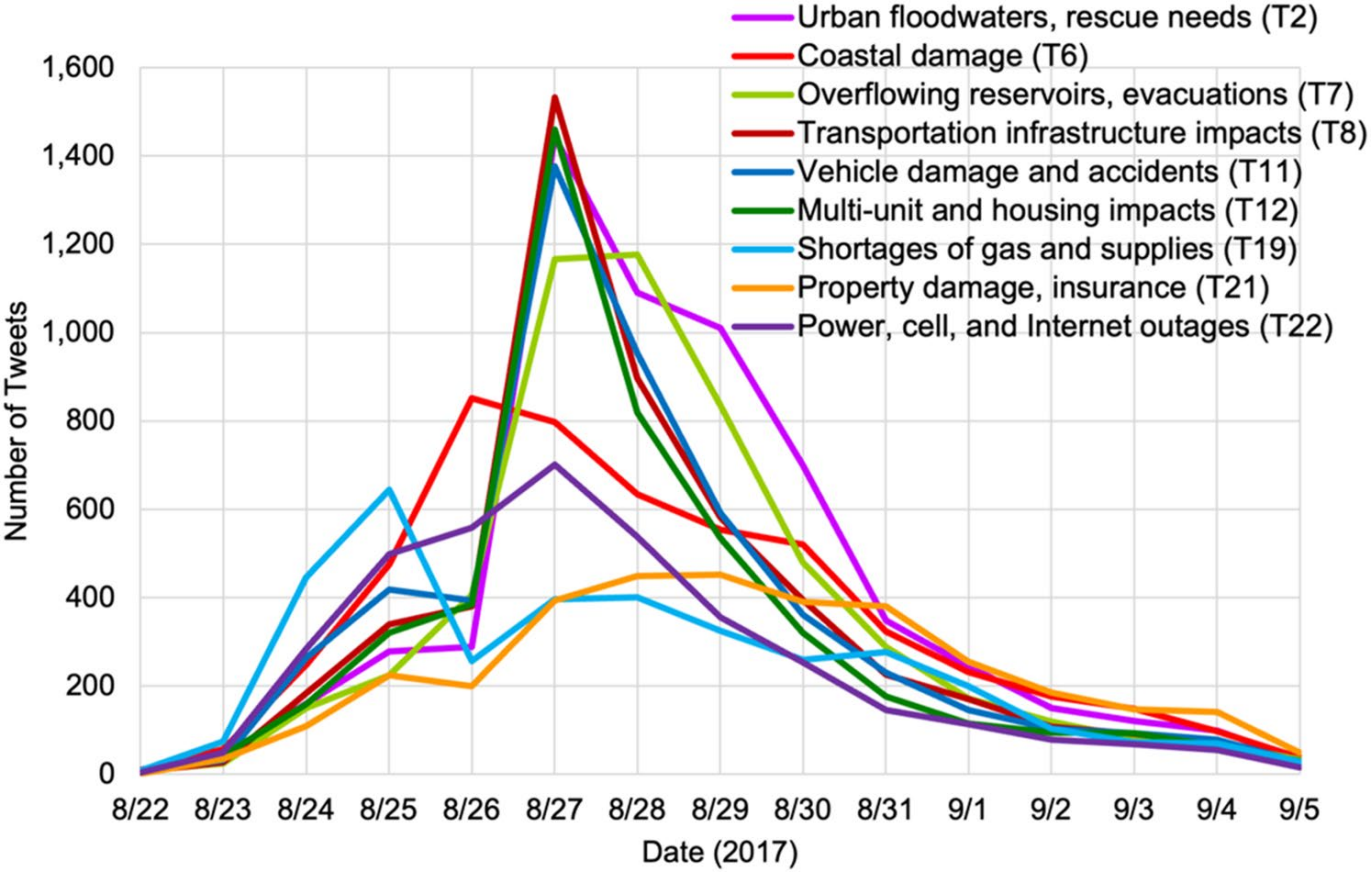
Timeline of topic frequencies. 25 August is the day of landfall, and 27 August corresponds to flooding in Houston MSA (metropolitan statistical area).

Figure 4 shows the volume of tweets in each topic across the timeline from the initial hurricane warning to landfall, to post-disaster recovery. We found that in the days preceding the landfall (25 August), shortages of gas and supplies (T19) were dominating the discussions. These discussions decreased substantially on the day of landfall, as many people were sheltering in place and the businesses were closed; also, the topic of coastal impacts (T6) came to forefront. On 27 August, we observed peak discussions of impacts to roads and transportation infrastructure (T8), impacts to housing and from the people who live in apartments and multiunit structures (T12), and damage or accidents involving personal vehicles (T11). This coincides with the peak impacts of flooding in urban areas, such as Houston and Beaumont. Moreover, Twitter users posted messages of both being and not being impacted. For example, there were messages about loss or no loss of power in T22, and similarly the floor being flooded or no structural damage in T12. Topics T2 of rescue needs, and T7 of evacuations due to flooded lakes and rivers, continued to be discussed at a higher rate after the Houston flooding, even after other topics started to subside.

The loss of electricity, cell, and Internet service (T22) did not exhibit a high peak but showed increased activity both on the day of the landfall and the next day when the flooding impacted Houston, and somewhat coincides with the topic of coastal damage (T6). This shows the importance of infrastructure resilience. Should there be a complete and total outage of all utilities, no such reports or communications would be possible. Finally, the topic of personal property damage and insurance claims (T21) slowly gained traction after the initial impacts of the disaster dissipated.

### Spatial Patterns and Correlation Analysis

**Fig. 5:**
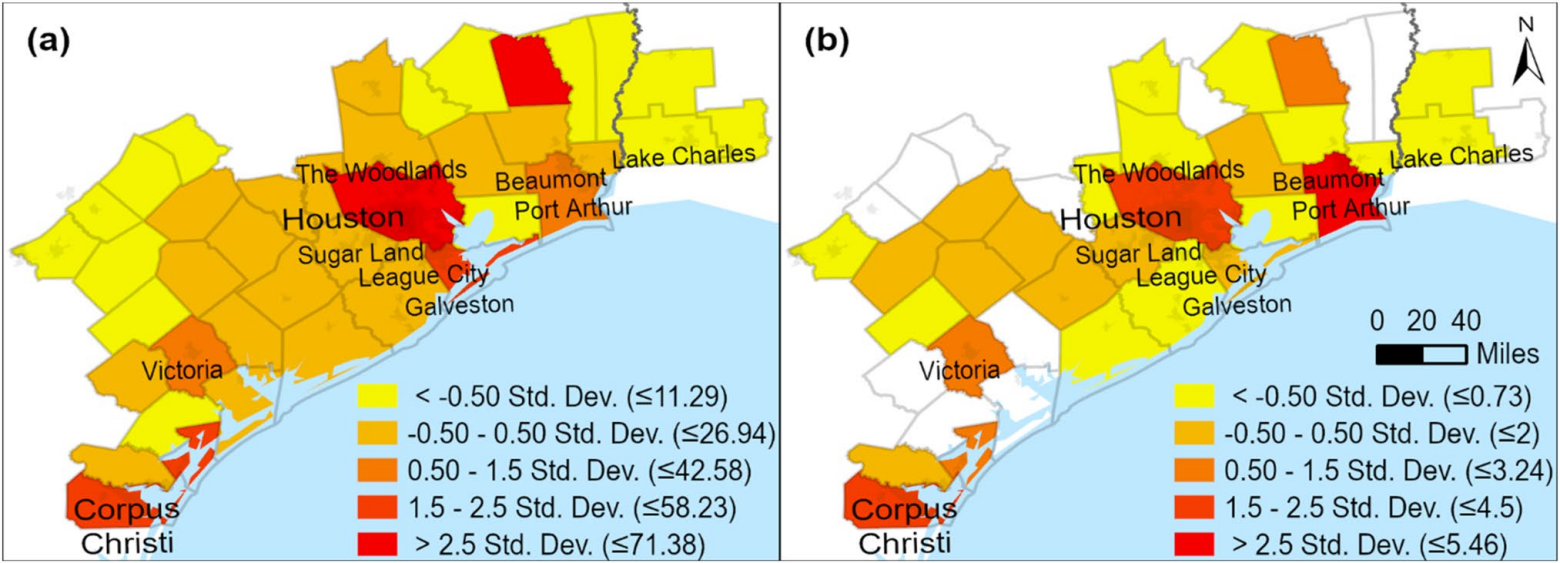
County map of **a** infrastructure related tweets and **b** tweets with images per 10,000 population

Figure 5 displays the spatial pattern of tweets as a sum of nine infrastructure-related topics in each county per 10,000 of their 2017 population. The counties with the most infrastructure related tweets were found to be Nueces and Aransas (Corpus Christi, TX), Harris and Galveston Counties, and Tyler County to the northeast of Houston. The pattern is similar to the pattern of tweets with images, except that the highest frequency of images was found in Jefferson County, TX, where Beaumont and Port Arthur are located.

To better understand this pattern, we tabulated the totals of tweets and useful images in each of the nine topics by county, normalized by population, and correlated them with the 3 real-world indicators: flood depths, damage per capita, and population density (Table 3). The maps of flood depth and damage are shown in Fig. 1a and b.

**Table 3:** Descriptive statistics of tweets and tweets with images per topic and their Pearson correlations with flood depth, damage per capita, and population density

Variables		Correlations			Descriptive statistics				
		Flood depth	Damage per capita	Population density	*N*	Min	Max	Mean	Std. Dev.
Average flood depth (in)		1	−0.47**	−0.282	36	3.88	30.56	12.93	7.77
Damage (USD per Capita)		−0.47**	1	−0.031	36	14.25	2973.93	524.6	671
Population density (per km^2^)		−0.282	−0.031	1	40	3.37	982.87	66.4	160.3
Tweets total***		−0.293	0.149	0.59**	40	0.7	71.38	19.11	15.84
Tweets with images total***		−0.296	0.164	0.333	28	0.21	5.46	1.36	1.28
Tweets by topic***:									
Floodwaters, rescue needs (2)	All	−0.082	0.013	0.54**	37	0.28	11.19	2.78	2.6
	With images	0.388	−0.082	−0.066	15	0.07	1.86	0.4	0.46
Coastal damage (6)	All	−0.196	0.183	0.155	39	0.21	20.2	3.81	4.69
	With images	0.256	−0.232	−0.114	14	0.05	2.57	0.53	0.67
Evacuations near waterbodies (7)	All	−0.163	−0.124	0.59**	37	0.32	8.73	2.61	1.99
	With images	−0.015	0.002	0.051	18	0.05	0.97	0.4	0.29
Transportation impacts (8)	All	−0.267	0.166	0.52**	36	0.48	9.32	2.4	2.22
	With images	−0.233	0.145	0.142	16	0.07	1.89	0.38	0.44
Vehicle damage and accidents (11)	All	−0.369*	0.231	0.75**	36	0.21	9.11	2.16	1.67
	With images	0.352	0.271	0.013	9	0.02	0.49	0.23	0.16
Multiunit and housing impacts (12)	All	−0.266	0.186	0.60**	36	0.32	8.35	1.98	1.84
	With images	0.058	0.015	0.578	10	0.04	0.5	0.18	0.16
Shortages of gas, supplies (19)	All	−0.260	−0.023	0.57**	34	0.27	6.45	1.63	1.45
	With images	0.138	0.112	−0.161	15	0.06	0.55	0.22	0.17
Property damage, insurance (21)	All	−0.276	0.144	0.61**	37	0.21	5.87	1.56	1.33
	With images	−0.171	0.81*	−0.306	8	0.02	0.81	0.17	0.27
Power, cell, Internet outages (22)	All	−0.289	0.17	0.55**	39	0.12	6.43	1.73	1.49
	With images	−0.030	0.70*	−0.267	11	0.04	1.21	0.24	0.35

^***^Per 10,000 population; **Correlation is significant at the 0.01 level (2-tailed); *Correlation is significant at the 0.05 level (2-tailed).

The results show significant correlation between the total number of tweets and population density, both overall and in each individual topic except the topic of coastal damage (T6). This is likely due to these topics being equally relevant to everyone affected by the disaster, thus making the spatial pattern of the infrastructure-related sample more driven by where people live. The exception of the topic of coastal impact (T6) supports this interpretation, as the topic mostly discussed impacts of the first landfall on the coastal areas, which would not be relevant to all counties with high population. In addition, significant negative correlation was found between the topic of vehicle damage and accidents (T11) and flood depth. This peculiarity can be explained by a reverse relationship of flood depth and damage in the study area (Fig. 1a, b). This is due to the less populated counties at the north of the study area containing more waterbodies and flooding, thus showing significantly higher average flood depth but with lower damage. This relationship is confirmed by the significant negative correlation between damage and flood depth. Thus, the negative correlation between vehicle damage topic and flood depth confirms that the spatial pattern of the topic resembles that of damage per capita.

Tweets with images do not have significant correlations with flood depth, damage, or population, which are likely due to irregularities introduced by the smaller sample size, with two notable exceptions. Images in the topics of property damage and insurance (T21) and prolonged outages (T22) exhibit significant correlation with the damage variable. The significant correlation between T21 and the damage variable is especially notable because the latter is represented by the FEMA disaster assistance amounts and people probably need photos for assistance claiming.

## Discussion

This study investigated the suitability of social media data for rapid infrastructure damage and impacts evaluation. More specifically, we used text mining and object recognition tools to estimate the volume of useful data present and their quality, such as how they can be used. For reliable damage assessments, it is desirable that data are collected timely, and have high spatial resolution and accurate description of the impact and/or photographic evidence. Ideally, this type of data would be uniform and machine readable to allow for quick aggregation and analysis. Based on these criteria, our assessment shows that Twitter data have advantages, as well as present a set of challenges that need to be overcome. One of the obvious advantages is that they are a real-time stream, which means that they can be used before any other field assessments can start safely. Another advantage is that they are crowd-sourced and sometimes contain detailed information that is relevant, such as describing impacts on specific locations or items of infrastructure. In addition, the data contain photographs that “zoom in” onto a particular impact, which can be used to supplement satellite or unmanned aerial vehicle (UAV) images. While we applied an image classification approach that is limited to one category per image, these photos or videos can be analyzed subsequently using computer vision algorithms for object recognition and damage quantification, such as water levels (Jafari et al. 2021), road washouts, landslides, bridge scouring, and downed power lines.

On the other hand, the challenge of coarse or inaccurate location information of Twitter data remains difficult to overcome. The location of a tweet can be inferred from the user’s profile location or from the location mentioned in the tweet (Middleton 2018). Having to infer location from free-form text is an example of Twitter data being unstructured, and presents a significant challenge, especially given that users often choose to input vague, broad reaching toponyms in their profile location to protect their privacy (Wang et al. 2021). Perhaps, since the original function of geotagging is no longer available, a compromise can be reached by allowing users attach accurate locations, such as coordinates, to their tweets at the time of major disasters to help emergency management making timely impact assessments.

Another challenging aspect is the impacts being presented as free form descriptions, mixed with other information of varying degree of relevance to the task of damage assessment. We approached this issue by applying a set of filtering steps and a topic modeling method to identify useful tweets and assign them with descriptive topics. These steps are taken as a “divide and conquer” strategy to navigate the huge volume of data more efficiently. The nine topics that we identified as useful to damage assessment represent different aspects of disaster impacts. However, to extract quantified impacts, further research and more sophisticated text mining techniques are needed. Similarly, image recognition narrowed down the data sample to most likely to be useful, but further development of object recognition models tailored to identify types and degree of damage from user-posted photographs would greatly improve the results.

Additionally, the topics identified in this study each can become subjects of focused research effort. For example, Khan et al. (2020) investigated supervised learning to detect transportation-related events from Twitter data (non-disaster related), whereas Chen and Ji (2021) used topic modeling to infer Hurricane Irma power outages from Twitter data. Our findings demonstrate that suitable social media data exist for many more applications, including flash floods and flooding reports, shortages of goods, vehicle accidents, housing damage, among others. The close matching of temporal patterns of the topics to real-world events suggests a possibility for successful event detection applications. On the other hand, the spatial patterns of the infrastructure-related tweets show correlation with population density but not distributed disaster aid (proxy for damage) or flood depth, suggesting that more research is needed to enable the use of Twitter data as a reliable spatial predictor in disaster modeling.

## Conclusion

This study analyzed the content of Twitter data collected during Hurricane Harvey to identify the data of the highest relevance for assessing the impacts on infrastructure through automatically grouping the tweets by topics of discussion. More specifically, we aimed to answer three research questions: (1) What are the common themes of discussion on Twitter during a major disaster, and do they contain infrastructure-related information? (2) How does the volume of tweets in each of the topics related to infrastructure impacts change over the course of the disaster response? (3) Does the spatial pattern of the locations of infrastructure-related tweets correlate with other measurements of real-world phenomena, such as flood depth, distributed disaster aid, or population density?

Through a series of filtering by keywords and geographic information and applying latent Dirichlet allocation modeling, we identified 24 topics that dominated Twitter during Hurricane Harvey. Among these topics, nine of them were of interest to this study. To answer the first research question, we found that the nine infrastructure-related themes were (1) urban flooding and needs for rescue vehicles; (2) impacts to coastal areas; (3) overflowing waterbodies and associated evacuations; (4) impacts to roads, highways, and airports; (5) personal vehicle impacts and road accidents; (6) impacts to multiunit housing; (6) shortages of gas and supplies; (7) personal property damage; (8) insurance claims; and (9) prolonged power, cell, and Internet outages. To answer the second question, we found that the relevance of the topics changed over time, with shortages of gas and supplies discussed primarily before the landfall, various damage impact topics during the active flooding phase, and the property damage and insurance claims gaining traction after the initial impacts dissipated. As for the third research question, we found significant correlations between the number of infrastructure related tweets and population density, whereas correlations with flood depth or disaster aid were not significant. This suggests that more research is needed to test if social media can be used as a reliable predictor to derive real-world estimates such as damage and flood depth.

Our study confirms that useful infrastructure-related messages in the Twitter data are mixed with other information, and a hierarchical strategy of filtering and LDA topic modeling allow for automatic and efficient grouping of the tweets based on their content. While previous studies identified the infrastructure-related messages among other categories in the Twitter data during major natural hazard-related disasters, our study expands on what kind of infrastructure-related information is posted. The results of this study will serve as baseline information for future research in social media text mining and object recognition from multimedia for infrastructure damage assessment.

## Footnotes

1 “airport, bayou, bridge, brownfield, bus, canal, cell, cleanup, communication, congestion, creek, culvert, dam, damage, debris, electricity, emergency management, emergency plan, energy, evacuate, evacuated, evacuating, evacuation, ferry, flood, floodwall, gas, general impact, hazardous, heating, highway, home, hospital, infrastructure, internet, interstate, landfill, levee, lock, oil, pavement, pipeline, port, power, power outage, rail, rebuild, repair, river, road, school, sewer, storm, stream, subway, surge, tanker, traffic, train, transportation, tree, utilities, waste, wastewater, water, waterway, wave, wind”

2 “SMART” dictionary from *tm* package; “amplification.words, deamplification.words, BuckleySaltonSWL, function.words, interjections, negation.words, Onix, TxtRetToolkitSWL1, preposition” dictionaries from *qdapDictionaries* package
